# On the origin of vertebrate somites

**DOI:** 10.1186/s40851-015-0033-0

**Published:** 2015-11-26

**Authors:** Takayuki Onai, Toshihiro Aramaki, Hidehiko Inomata, Tamami Hirai, Shigeru Kuratani

**Affiliations:** Kuratani Evolutionary Morphology Laboratory, RIKEN Center for Developmental Biology, 2-2-3 Minatojima-Minamimachi, Chuo-ku Kobe, 650-0047 Japan; Pattern Formation Group, Graduate School of Frontier Biosciences, Osaka University, 1-3 Yamadaoka, Suita, Osaka 565-0871 Japan; Laboratory for Axial Pattern Dynamics, RIKEN Center for Developmental Biology, 2-2-3 Minatojima-Minamimachi, Chuo-ku Kobe, 650-0047 Japan

**Keywords:** Amphioxus, Somites, Notch signalling, Origin of vertebrates, Enterocoel theory

## Abstract

**Introduction:**

Somites, blocks of mesoderm tissue located on either side of the neural tube in the developing vertebrate embryo, are derived from mesenchymal cells in the presomitic mesoderm (PSM) and are a defining characteristic of vertebrates. In vertebrates, the somite segmental boundary is determined by Notch signalling and the antagonistic relationship of the downstream targets of Notch, Lfng, and Delta1 in the anterior PSM. The presence of somites in the basal chordate amphioxus (*Branchiostoma floridae*) indicates that the last common ancestor of chordates also had somites. However, it remains unclear how the genetic mechanisms underlying somitogenesis in vertebrates evolved from those in ancestral chordates.

**Results:**

We demonstrate that during the gastrula stages of amphioxus embryos, Bf*Fringe* expression in the endoderm of the archenteron is detected ventrally to the ventral limit of Bf*Delta* expression in the presumptive rostral somites along the dorsal/ventral (D/V) body axis. Suppression of Notch signalling by DAPT (a γ-secretase inhibitor that indirectly inhibits Notch) treatment from the late blastula stage reduced late gastrula stage expression of Bf*Fringe* in the endodermal archenteron and somite markers Bf*Delta* and Bf*Hairy-b* in the mesodermal archenteron. Later in development, somites in the DAPT-treated embryo did not separate completely from the dorsal roof of the archenteron. In addition, clear segmental boundaries between somites were not detected in DAPT-treated amphioxus embryos at the larva stage. Similarly, in vertebrates, DAPT treatment from the late blastula stage in *Xenopus* (*Xenopus laevis*) embryos resulted in disruption of somite Xl*Delta-2* expression at the late gastrula stage. At the tail bud stage, the segmental expression of Xl*MyoD* in myotomes was diminished.

**Conclusions:**

We propose that Notch signalling and the Fringe/Delta cassette for dorso-ventral boundary formation in the archenteron that separates somites from the gut in an amphioxus-like ancestral chordate were co-opted for anteroposterior segmental boundary formation in the vertebrate anterior PSM during evolution.

**Electronic supplementary material:**

The online version of this article (doi:10.1186/s40851-015-0033-0) contains supplementary material, which is available to authorized users.

## Introduction

Segmented structures composed of repetitive units, called somites, that arise transiently during embryogenesis are a key feature of the vertebrate body plan. The somites lie laterally to the notochord, and a spinal nerve forms a segmental unit assigned to somitic derivatives in the trunk [1, 2]. During development, somites differentiate into myotomes and skeletal elements that form the basic supporting structure in adults. From an evolutionary perspective, somites are conserved between vertebrates and amphioxus but are secondarily lost in tunicates [3–5]. Amphioxus (*Branchiostoma floridae*), also known as cephalochordate or lancelet, has a notochord, neural tube, and pharynx, but lacks a neural crest and placodes. In amphioxus, somites extend into the anterior end, unlike in vertebrates, and amphioxus is thus considered not to possess a homolog of the vertebrate unsegmented head mesoderm; however, this contention remains controversial (Additional file 1: Figure S1) [6, 7]. A recent genome analysis indicated that amphioxus retains most of the developmental genes, such as Hox clusters, present in vertebrates and is thus the best proxy to address the origin of the vertebrate body plan [8].

**Fig. 1:**
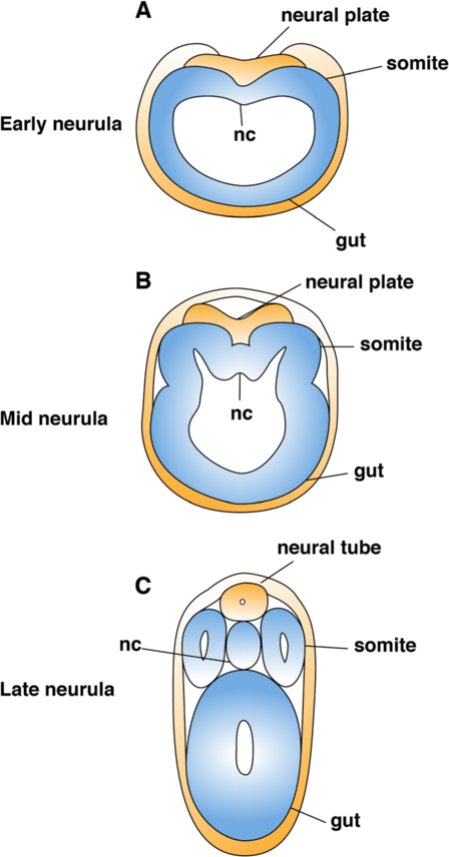
Development of amphioxus rostral somites. **a** At the early neurula stage, the dorsal roof of the anterior archenteron begins to expand dorsolateral to the ectoderm. **b** At the mid-neurula stage, the rostral somites swell and form a U-shape that remains part of the dorsal roof of archenteron. **c** At the late neurula stage, the somites pinch off from the archenteron roof. *nc* notochord

Although somites are common in chordates, there are some differences in developmental sequences between amphioxus and vertebrates. For example, in amphioxus, the rostral somites develop from the dorsal mesoderm during early embryogenesis. The mesoderm is a single layer located in the dorsal roof of the archenteron (endodermal/mesodermal structure) at the early neurula stage (Fig. 1a). By the mid-neurula stage, a prospective somite swells dorsolaterally and the bottom narrows (Fig. 1b). At the late neurula stage, all the rostral somites pinch off from the archenteron roof simultaneously and line up laterally to the notochord (Fig. 1c). Beginning from the late neurula stage, the caudal somites bud off one by one directly from the lateral epithelial cells in the tail bud by schizocoely (a coelom is formed by separating mesenchymal cells) [9]. Whereas, in vertebrates, somites are formed in pairs in the anterior end of the PSM through gradual epithelialization [10]. These developmental differences raise the question of the ancestral origin of somitogenesis.

Somites do not occur in any group other than chordates. However, a previous study indicated that the evolutionary origin of somites could be found in cnidarians, diploblastic animals that are thought to be ancestral to the bilaterians [11]. According to Sedgwick’s enterocoel theory, chordate somites are derived from the alimentary pouches of coelenterates (ctenophores and cnidarians) [11]. Nonetheless, it is still debated whether coelomic cavities are ancestral to all bilaterians (e.g., the basal bilaterian Acoela does not have coelomic cavities), as there is no persuasive evidence to support this hypothesis [12–14]. The enterocoel theory promotes the view that the rostral somites are more ancestral than the caudal somites in amphioxus and the vertebrate somites. However, without grounding in molecular genetic comparisons, the somite evolutionary scenario remains enigmatic.

Recent molecular studies have revealed the developmental mechanisms underlying somitogenesis. In vertebrates, somites are formed by several developmental sequences. Starting from gastrulation, the PSM is internalized around the blastopore and becomes part of the tail bud at the posterior end of the body. A future pair of somites is gradually epithelialized in the anterior PSM, and a boundary between each somite is determined. One pair of somites is formed every 120 min in mice and every 25 min in zebrafish, indicating the interspecies differences in somitogenetic rhythms among vertebrates [15]. Molecular oscillators control this rhythmic somitogenesis. Of these, Notch signalling is a key factor in the anterior PSM, or “determination front”, and is essential for the boundary formation of future segments [10]. At the determination front, the lunatic fringe (*Lfng*) glycosyltransferase, a downstream modulator of Notch signalling, has been reported to be essential for generating a boundary by modifying the Notch receptor in mouse embryos [15]. Additionally, in zebrafish, *Lfng* is expressed in the tail bud where, unlike in mouse and chick embryos, expression does not oscillate, suggesting a diverse role for somitogenesis, which may have been secondarily lost in the anole lizard, as *Lfng* is not expressed in the PSM in lizard embryos [16, 17]. Upstream of *Lfng*, mesoderm posterior (*Mesp2*), a member of the basic helix-loop-helix transcriptional factor family, regulates Notch signalling input [18].

In amphioxus, the dorsal mesoderm includes somites and the notochord the initial somite boundary formation is recognized in the dorsal mesoderm at the mid-gastrula stage. A pair of stripes of Bf*Delta* (the homolog of DLL1) expression is detected in the first somites [19]. Additionally, several homologs of vertebrate segmentation genes, such as *Hairy* and *Uncx4.1*, are expressed in somites from the gastrula to the larval stages [9]. However, no direct evidence of periodic expression of cyclic genes in the tail bud has been reported. Although the amphioxus genome harbours a *Mesp* homolog, it is not likely to be expressed during embryogenesis [9]. Bf*Fringe* is expressed in the neural tube and the endodermal gut, but not in the somites, during the neurula stages [20]. Evidence suggests that the periodic expression of cyclic genes and the establishment of the determination front by the *Mesp* and *Lfng* genes are vertebrate-specific genetic networks.

In this study, we investigated the molecular mechanisms underlying somitogenesis in amphioxus embryos with a focus on Notch signalling. Since current evidence suggests that vertebrate somites evolved from somites of an amphioxus-like ancestral animal, we performed gene expression and functional analyses using amphioxus and *Xenopus* embryos.

## Materials and methods

### Collection of amphioxus embryos

Amphioxus adults were collected in Old Tampa Bay, Florida, USA during the summer breeding season in 2011. The animals were subjected to electric shock in filtrated seawater at night (21:00 to midnight), as described previously [21], to promote spawning. The fertilized eggs were cultured in culture dishes (6–36 h) with filtrated seawater at room temperature.

### Xenopus experiments

Following *in vitro* fertilization, the embryos were cultured in 0.1× Barth’s solution until DAPT (Tocris, Bristol, UK) treatment. Staging was performed based on the normal table of Nieuwkoop and Faber [22].

### DAPT treatments

DAPT was dissolved in DMSO (100 mM). Fifty [23] to 100 micromolar DAPT was added to amphioxus embryos at the late blastula stage at room temperature. The larval morphology of 50–100 μM DAPT-treated embryos was similar.

The same amount of DMSO was applied to the seawater as a control. The DAPT-treated embryos were further cultured until they reached the late gastrula or larval stages for fixation. In *Xenopus*, 200 μM DAPT or the same amount of DMSO for controls was added to 0.1× Barth’s solution at the late blastula stage, and the embryos were further cultured until fixation. One hundred micromolar DAPT treatment in *Xenopus* did not lead to any overt phenotypes, as seen in Fig. 6. Embryos were fixed in 4 % paraformaldehyde in 3-(N-morpholino) propanesulfonic acid (0.1 M) solution for amphioxus or in 3-(N-morpholino) propanesulfonic acid (0.1 M)/2 mM EGTA/1 mM magnesium sulphate/3.7 % formaldehyde solution for *Xenopus* embryos. Amphioxus embryos were stored in 70 % EtOH, and *Xenopus* embryos were stored in 100 % MeOH until further analysis.

### Whole-mount in situ hybridization (WISH) and F-actin staining

WISH and F-actin staining with BODIPY FL phallacidin (Molecular Probes, Minato, Tokyo, Japan) were performed following previously published protocols [23–25]. WISH was performed for the following genetic markers: Bf*Delta* [19]*,* Bf*Hairy-b* [26]*,* Bf*Gsc*, Bf*Bra* [21], Bf*Muscle-actin* [25], Bf*Fringe* [20], Bf*Mrf1* [27], Xl*Gsc* [28], Xl*Bra* [29], Xl*Delta2* [30], Xl*MyoD* [31], and Xl*Tbx1* [32]. Zeiss LSM 710 or LSM 780 confocal microscope (Zeiss, Shinjuku, Tokyo, Japan) was used for detecting *F-Actin* signals and fluorescence WISH.

### Embedding and sectioning of amphioxus embryos

Following WISH, the embryos were washed in distilled water for 3 min. The embryos were stained with Ponceau S solution (SIGMA, Shinagawa, Tokyo, Japan), incubated for 1 h, and then washed in an EtOH series (75–100 %). After removal of the EtOH, resin (Polysciences, Inc., Warrington, PA, USA) was added to the dishes and incubated for 30 min. Samples were transferred to an embedding mould and treated with fresh resin for 30 min. The mould was then placed in an oven (67 °C overnight). The embedded samples were cut with a sharp glass knife, and the sections were carefully transferred to glass slides.

## Results

### Dorso-ventral boundary formation between the rostral somites and the gut is controlled by Notch signalling

**Fig. 2:**
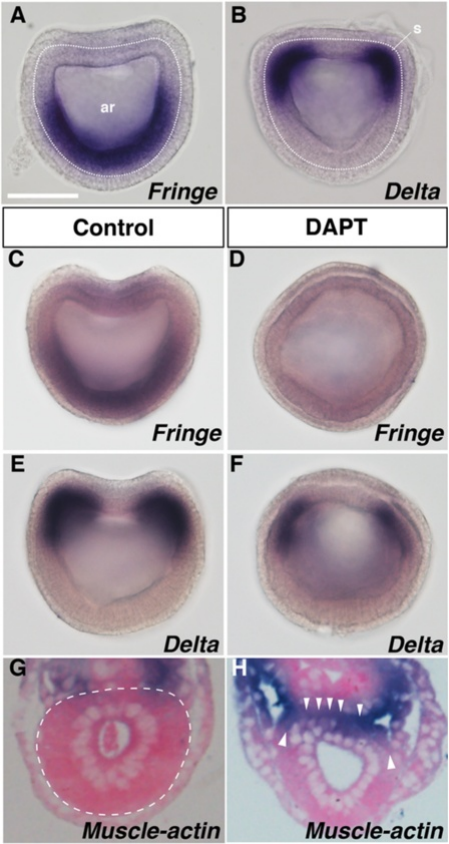
Notch signalling controls the pinching off process of the rostral somites. **a**–**b** Bf*Fringe* expressed in the anterior endoderm (ventral part of the archenteron) and Bf*Delta* was expressed in the presumptive rostral somites at the late gastrula stage. Blastopore views with the dorsal side up. The white dotted circle indicates the archenteron. *s* somite, *ar* archenteron. Scale bar, 50 μm. **c**–**f** Effect of 100 μM DAPT treatment on Bf*Fringe* (*n* = 7, 100 %) or Bf*Delta* (*n* = 8, 100 %). Anterior views with the dorsal side up. **g** In the DMSO-treated control larval embryo, the segmental boundary between the somite and the dorsal gut roof was clear (*n* = 1). **h** In larval embryos treated with DAPT, the boundary was unclear and ectopic expression of *Muscle-actin* was observed (*n* = 1). Transverse sections with the dorsal side up. White arrowheads indicate somite and gut fusion locations. The white dotted circle indicates the border between the gut and somites

In vertebrates, Lfng suppresses Notch signalling in the anterior PSM and controls segmental boundary determination of future somites [33]. In amphioxus, Bf*Fringe* is expressed in the neural plate but not in the somites or tail bud [20]. However, at the late neurula stage, Bf*Fringe* is expressed in the ventral part of the dorso-ventral boundary between somites and the gut, suggesting that Bf*Fringe* regulates dorso-ventral boundary formation of the archenteron [20]. We examined the expression pattern of Bf*Fringe* at the late gastrula stage and found that it was expressed in the ventral half of the archenteron (Fig. 2a). The dorsal limit of Bf*Fringe* expression was located ventral to Bf*Delta* expression in the presumptive rostral somites (Fig. 2b). These findings suggest that the Fringe/Delta cassette controls dorso-ventral boundary formation between the mesoderm (somites) and the endoderm (gut) in the archenteron.

In vertebrates, Notch signalling regulates *Lfng* expression in the PSM. For example, DAPT treatment in chick embryos affects the cyclic expression of *Lfng* [34]. To test whether Notch signalling controls the expression of Bf*Fringe* and Bf*Delta* in amphioxus embryos, we treated the embryos from the late blastula stage with 100 μM DAPT. DAPT-treated embryos displayed severe defects in the pinching off process during somitogenesis. At the very late gastrula stage, DMSO-treated control embryos exhibited swelling of the lateral dorsal roof of archenteron, while morphological transformation of the dorsal archenteron roof was not detected in the DAPT-treated embryos (Fig. 2c and d). Furthermore, the expression of Bf*Fringe* was eliminated from the anterior endoderm in DAPT-treated embryos (Fig. 2c and d). In contrast, Bf*Delta* expression was detected in the presumptive somites both in the control and DAPT-treated embryos, but the expression was reduced in the latter (Fig. 2e and f). Later in development, DAPT treatment expanded the localization of expression of Bf*Muscle-actin* (a conserved structural protein in muscles expressed in myotomes) medially into the gut at the larval stage, and the boundary between the somites and the gut became vague (Fig. 2g and h). These results indicate that Notch signalling and the Fringe/Delta cassette are essential for dorso-ventral boundary formation between the somites and the gut.

### Notch signalling is essential for rostral somite formation in amphioxus embryos

**Fig. 3:**
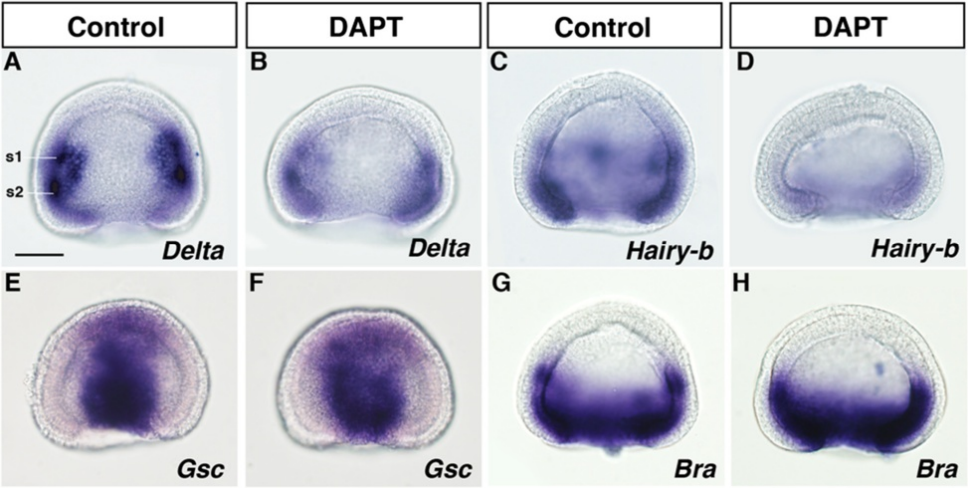
DAPT treatment affects somitogenesis in amphioxus embryos. Effect of treatment with 100 μM DAPT at the late blastula stage on mesodermal gene expression (**a**, **b** *Delta*; **c**, **d**, *Hairy-b*; **e**, **f**, *Gsc*; **g**, **h**, *Brachyury*; *n* = 10 each, 100 %*)* at the gastrula stage. Dorsal views with the anterior side up. s, somite. Scale bar, 50 μm (**a**)

**Fig. 4:**
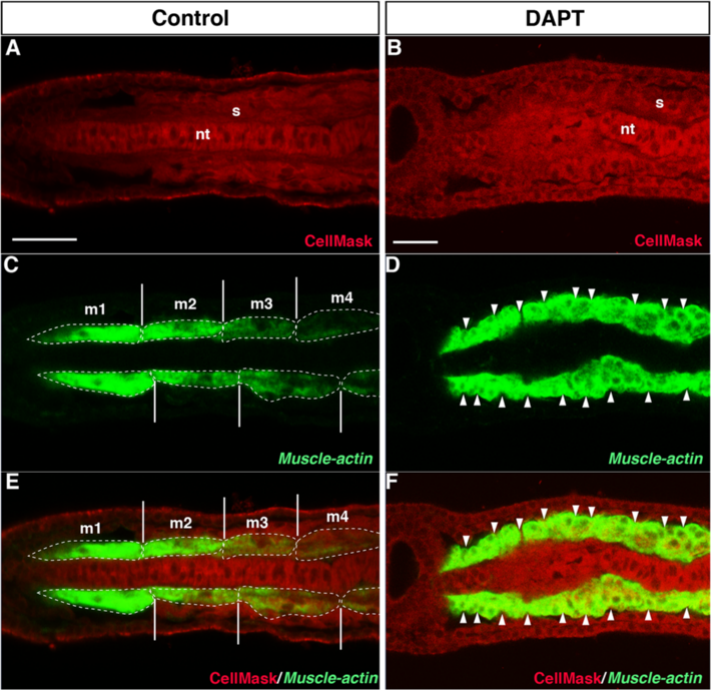
Effect of DAPT treatment on Bf*Muscle-actin* expression. **a**, **c**, **e** DMSO-treated control embryo (*n* = 5, 100 %). Anterior to the left. Dorsal view. nt, notochord; s, somite; m, myotome. CellMask (*Red*) labelled the plasma membrane. Bf*Muscle-actin* (*Green*) was stained by fluorescence *in situ* hybridization. The white dotted circle indicates a myotome. The white line indicates the segmental border between myotomes. **b**, **d**, **f** DAPT-treated embryo (*n* = 5, 100 %). Anterior to the left. Dorsal view. The white arrowhead indicates a possible segmental border between myotomes. Scale bars, 20 μm

The essential role of Notch signalling on formation of the dorso-ventral boundary between the gut and somites suggests that Notch signalling may also play a role in segmental boundary formation between somites in amphioxus embryogenesis. To address this questions, we studied the role of Notch signalling in segmental boundary formation between somites. In amphioxus, the first and second somites express the somite markers Bf*Delta* and Bf*Hairy-b* at the late gastrula stage [19, 26]. Treatment with 100 μM DAPT from the late blastula stage reduced Bf*Delta* and Bf*Hairy-b* expression (Fig. 3a–d), indicating that Notch signalling is essential for rostral somite formation. Treatment with DAPT had little effect on the expression of the axial mesoderm marker Bf*Gsc* and the pan-mesoderm marker Bf*Bra* (Fig. 3e–h). These findings suggest that the role of Notch signalling is to specify the rostral somites rather than to induce the entire dorsal mesoderm. To determine whether the effect of DAPT treatment disrupts segmental boundaries in the rostral somites, we examined later stage phenotypes. At the larval stage, DAPT-treated embryos displayed severe morphological abnormalities; both notochord and somites bent mediolaterally and epidermal cells were much thicker than those of the control embryos (Fig. 4a and b). In control embryos, Bf*Muscle-actin* expression was detected in myotomes that were asymmetric along with the left/right (L/R) body axis (Fig. 4c and e). In DAPT-treated embryos, Bf*Muscle-actin* expression was observed in myotomes; however, the size of each myotome was smaller and the boundaries between myotomes were less clear compared with those of the control embryos. In addition, the asymmetry of somites was disturbed in DAPT-treated embryos (Fig. 4d and f) [35, 36].

**Fig. 5:**
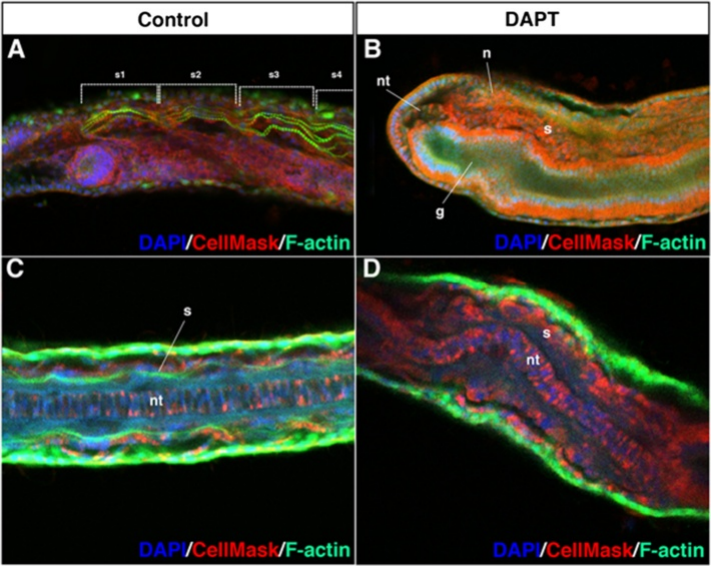
Filament formation of actin is suppressed by DAPT treatment. **a** DMSO-treated control embryos display filamentous actin in the rostral somites (*n* = 12, 100 %). **b** F-actin staining in the somites. Treatment with 100 μM DAPT from the late gastrula stage onwards (*n* = 10, 100 %). The images were taken using an LSM 710 confocal microscope (Zeiss). **c** Dorsal view of DMSO-treated control embryos. **d** Dorsal view of DAPT-treated embryos. *s* somite, *nt* notochord, *n* neural tube, *g*, gut. Nuclei were labelled in blue and the plasma membrane was labelled in *red*

The incomplete formation of the segmental boundary of each somite in the DAPT-treated embryos prompted us to test whether actin filament formation was normally organized in the somites. In the DMSO-treated control embryos, three or four muscle fibres were formed in each somite (Fig. 5a). However, the DAPT-treated embryos did not have actin filaments (no F-actin signal was detected in the somites; Fig. 5b–d). Together, these findings suggest that Notch signalling is essential for both segmental boundary formation of the somites and differentiation of muscle fibres. As Bf*Fringe* is not expressed in the somites, the Notch signalling-mediated regulation of segmental boundary formation between somites involves genetic networks distinct from that underlying dorso-ventral boundary formation between the gut and somites.

### Notch signalling is required for somitogenesis from the gastrula stages in vertebrate embryos

**Fig. 6:**
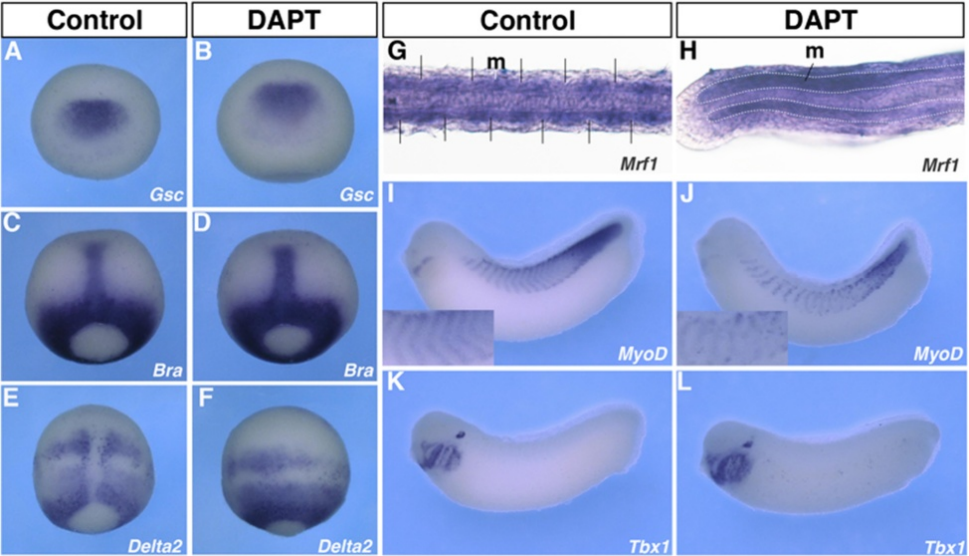
Inhibition of Notch signalling results in loss of segment formation in *Xenopus* embryos. **a**–**f** DAPT treatment did not affect the expression pattern of *Gsc* (**a** *n* = 42, 93 %; **b** *n* = 38, 87 %) and *Brachyury* (**c** *n* = 20, 95 %; **d** *n* = 14, 94 %), whereas it disrupted the paired-stripe expression pattern of *Delta-2* (**e** *n* = 33, 100 %; **f** *n* = 47, 51 %)*.* **a**–**b** Anterior views with dorsal side up. **c**–**f** Dorsal views with the anterior side up. **g**–**h** DAPT treatment resulted in loss of segmental expression of *Mrf1* in amphioxus (**g** *n* = 12, 100 %; **h** *n* = 11, 100 %. Dorsal views with anterior to the left), *MyoD* in *Xenopus* (**i** *n* = 23, 100 %; **j** *n* = 15, 93 %), and *Tbx1* (**k** *n* = 23, 100 %; **l** *n* = 13, 54 %). **i**, **j** The lower left panel displays the magnification of the somite expression of *MyoD*

Suppression of Notch signalling resulted in reduced somite marker expression in amphioxus at the late gastrula stage (Fig. 3a–d). In vertebrates, the dorsal mesoderm is regionalized into the head and trunk mesoderm during the gastrula stages, and the somite progenitor cells are generated in the trunk mesoderm [37]. However, it is unclear whether Notch signalling is crucial for the initial differentiation of somites during gastrula stages. To address this, we investigated the role of Notch signalling in somitogenesis during the gastrula stages. In *Xenopus,* Xl*Gsc* and Xl*Bra* were expressed in the head mesoderm and the notochord, respectively, while Xl*Delta-2* was expressed in the region of the trunk somites at the late gastrula stage (Fig. 6a, c, e). Similar to the findings in amphioxus embryos, treatment with DAPT from the late blastula stage did not affect the expression of Xl*Gsc* and Xl*Bra*, while Xl*Delta-2* expression was severely disrupted (Fig. 6a–f). This suggests that, as in amphioxus, Notch signalling regulates the formation of somites, but not mesoderm induction, during the gastrula stages.

Additionally, DAPT-treated *Xenopus* embryos exhibited severe phenotypes at the tail bud stage. Bf*Mrf1* is a homolog of the vertebrate *MyoD* gene, a key marker of early muscle differentiation [27]. Treatment of the embryos with 100 μM DAPT from the late blastula stage onwards resulted in loss of segmental Bf*Mrf1* expression in the somites (Fig. 6g and h). DAPT treatment had a severe effect on the segmental expression of Xl*MyoD* in the somites. In control embryos, Xl*MyoD* expression was observed in each somite in a V-shaped pattern. However, this V-shaped expression was disrupted, and the borders of Xl*MyoD* expression in each somite were indistinct in DAPT-treated embryos (Fig. 6i and j). In contrast, expression of the pharyngeal mesoderm marker Xl*Tbx1* was relatively normal in the DAPT-treated embryos (Fig. 6k and l). Taken together, these results suggest that Notch signalling controls somitogenesis from the gastrula stage in amphioxus and vertebrates.

## Discussion

### Somitogenesis in amphioxus embryos

Previous molecular studies of amphioxus somitogenesis revealed that although rostral and caudal somites display differences in their developmental processes, both express conserved somite segmentation markers (e.g., *Tbx15/18/22, Delta, Notch, Hey, HairyC, HairyD, IrxA, NeuroD, Ripply, Six1/2*). These are thus considered to involve similar developmental mechanisms despite the observation that some genes, such as Bf*En*, are expressed only in rostral somites [9, 38].

A functional study of Fgf signalling, however, demonstrated that loss of Fgf signalling affects only rostral and not caudal somite segmentation [39]. This suggests that the molecular mechanisms underlying somite segmental boundary formation and AP body axis formation in amphioxus are different. In this study, we demonstrated that Notch signalling is essential for dorso-ventral boundary formation between the somites and the gut in the archenteron. In addition, our data suggest that the Fringe/Delta cassette under Notch signalling is likely important in dorso-ventral boundary formation in the archenteron. Notch signalling plays also an essential role in segmental boundary formation between somites. We have also shown that DAPT treatment results in loss of the F-actin formation in myotomes. Finally, our data indicate that Notch signalling is important for the L/R asymmetry of somites. Current data suggest that Notch and Fgf signalling regulate the segmental boundaries of rostral somites, but it is unclear whether crosstalk between Notch and Fgf signalling exists in rostral somite segmentation.

In amphioxus, caudal somites develop directly from the tail bud. In the control larval embryos, segmental expression of Bf*Muscle-actin* was detected in caudal myotomes (Additional file 1: Figure S2A, C, E). Additionally, caudal myotomes were asymmetric (Additional file 1: Figure S2E). In DAPT-treated embryos, expression of Bf*Muscle-actin* was somewhat continuous, and clear segmental boundaries between somites were nearly undetectable (Additional file 1: Figure S2B, D, F). Furthermore, the asymmetric organization of somites along with the L/R body axis was irregular (Additional file 1: Figure S2F). These results indicate that Notch signalling plays an essential role in caudal somitogenesis. As Fgf signalling does not play a primary role in caudal somite segmentation, Notch signalling is probably the major factor driving segmental border formation in caudal somites.

### Gut pouches and the origin of the somites

**Fig. 7:**
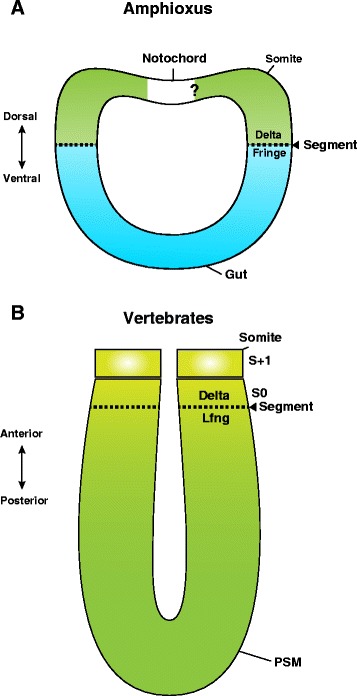
A new evolutionary scenario of the chordate somite formation. **a** In amphioxus, mesoderm/endoderm boundary formation is organized by Fringe/Delta cassette under Notch signalling. **b** The Notch signalling-dependent mechanism of segmental boundary formation in (**a**) was co-opted to the future segments in the anterior PSM of amniotes embryos. The mesoderm is presented in green. The endoderm is in light blue. The dotted line indicates a segmental border

Based on the results of the current study, we propose that Notch signalling-dependent segmental boundary formation between the gut and somites in an amphioxus-like ancestor may have been co-opted by vertebrates and used in segmental border determination between the propsective somites and the anterior PSM (Fig. 7). According to the enterocoel theory, the dynamic transformation of a part of the endoderm to mesoderm appears to have happened during evolution from a cnidarian-like diploblastic ancestor to triploblastic animals [11, 14, 40]. Recent molecular phylogeny proposed that deuterostomes are in a basal lineage of bilateria [41].

In amphioxus, the mesoderm and endoderm originate from the same single layer (archenteron), and the mesoderm differentiates into the musculature somites and the notochord, from which muscle fibres extend to the neural tube [42, 43]. A recent gene expression profiling study demonstrated that mesodermal genes are expressed in the embryonic endoderm of the sea anemone *Nematostella* (a member of the phylum Cnidaria) [44], suggesting that a new genetic program for mesoderm formation in endoderm evolved in Urbilateria. For the evolution of mesoderm in the archenteron, axial patterning events that occur during early embryogenesis could be important. In deuterostomes, dorsal/ventral (D/V) axial determination is regulated by members of the TGF-β super family, including *Nodal* and *Bmps* [21]*.* Nodal and Bmp antagonize each other to generate D/V polarity. Conserved Nodal/Bmp antagonism in deuterostomes is implicated in a fundamental genetic mechanism specifying and maintaining D/V polarity in mesoderm development [21]. Future studies will address how the mesoderm specification gene networks evolved in the archenteron by reorganizing the relationship between Nodal signalling and key mesodermal genes (e.g. *Gsc, Bra,* and *Twist*) and generating the mesoderm/endoderm boundary in the archenteron by Notch signalling. The genome of the ctenophore (*Mnemiopsis leidyi*) has many Notch signalling components, suggesting an ancient role for Notch signalling in boundary formation [45].

In hemichordates, a sister group of chordates, the longitudinal muscles run just underneath the dorsal nerve cord [46]. In some hemichordate species (e.g., *S. kowalevskii*), the mesoderm is derived from the endoderm by enterocoely (the mesodermal coelom forms by outpocketing of the gut) [46, 47]. Currently, it is unclear whether the genetic programs that underlie enterocoelic mesoderm formation are shared between hemichordates and amphioxus.

## Conclusions

Our results reveal that Notch signalling and the Fringe/Delta cassette regulates dorsal-ventral boundary formation in the archenteron to segregate somites from the gut in the basal chordate amphioxus. These findings increase our understanding of how the vertebrate body plan evolved by recapitulating ancestral developmental programs (e.g. segmental boundary formation in amphioxus archenteron) and adapting them to a novel developmental event (e.g. segmental border formation in vertebrate anterior PSM) as a heterotopic shift.

## Additional file

Additional file 1:
**Figure S1.** F-actin staining of adult amphioxus (*Branchiostoma japonicum*). In amphioxus, myotomes extend to the rostral end. Anterior to the left. nt, notochord; m, myotome; bc, buccal cirri; df, dorsal fin; pm, pterygeal muscle. **Figure S2.** Inhibition of Notch signalling and the effect on posterior myotomes at the early larva stage. (A, C, E) In the DMSO-treated control embryos, myotomes lay laterally to the notochord, and Bf*Muscle-actin* was expressed in the myotomes (*n* = 5, 100 %) (B, D, F) In the DAPT-treated embryos, Bf*Muscle-actin* expression was detected in the myotomes (*n* = 5, 100 %). The while line in (E) indicates the border of each myotome. The white dotted circle in (E) and (F) indicates a myotome or tail bud. The white arrowheads in (F) indicate a possible boundary between myotomes. m, myotome; nt, notochord; t, tail bud. Scale bars, 20 μm. (DOCX 2317 kb)
