# Supplementary material for: On the origin of vertebrate somites
Source: Zoological Lett. 2015 Nov 26;1:33. doi: 10.1186/s40851-015-0033-0 (PMC4660845; doi:10.1186/s40851-015-0033-0)
Supplement: Additional file 1: — Figure S1. F-actin staining of adult amphioxus (Branchiostoma japonicum). In amphioxus, myotomes extend to the rostral end. Anterior to the left. nt, notochord; m, myotome; bc, buccal cirri; df, dorsal fin; pm, pterygeal muscle. Figure S2. Inhibition of Notch signalling and the effect on posterior myotomes at the early larva stage. (A, C, E) In the DMSO-treated control embryos, myotomes lay laterally to the notochord, and BfMuscle-actin was expressed in the myotomes (n = 5, 100 %) (B, D, F) In the DAPT-treated embryos, BfMuscle-actin expression was detected in the myotomes (n = 5, 100 %). The while line in (E) indicates the border of each myotome. The white dotted circle in (E) and (F) indicates a myotome or tail bud. The white arrowheads in (F) indicate a possible boundary between myotomes. m, myotome; nt, notochord; t, tail bud. Scale bars, 20 μm. (DOCX 2317 kb) [file 40851_2015_33_MOESM1_ESM.docx]

**Supplementary information**

**On the origin of vertebrate somites**

**Takayuki Onai, Toshihiro Aramaki, Hidehiko Inomata, Tamami Hirai and Shigeru Kuratani**


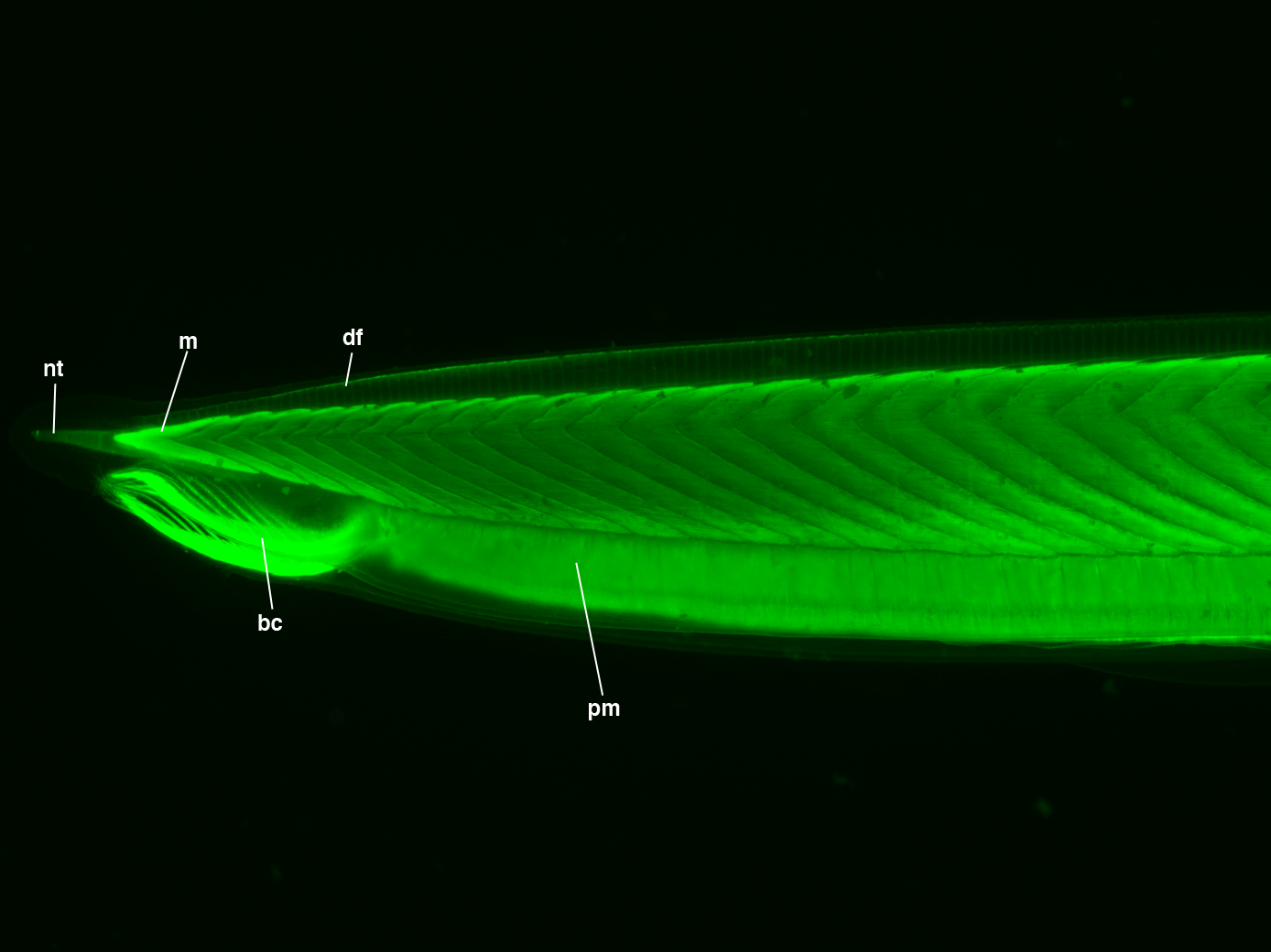


Supplemental Figure S1. F-actin staining of adult amphioxus (*Branchiostoma japonicum*).

In amphioxus, myotomes extend to the rostral end. Anterior to the left. nt, notochord; m, myotome; bc, buccal cirri; df, dorsal fin; pm, pterygeal muscle.


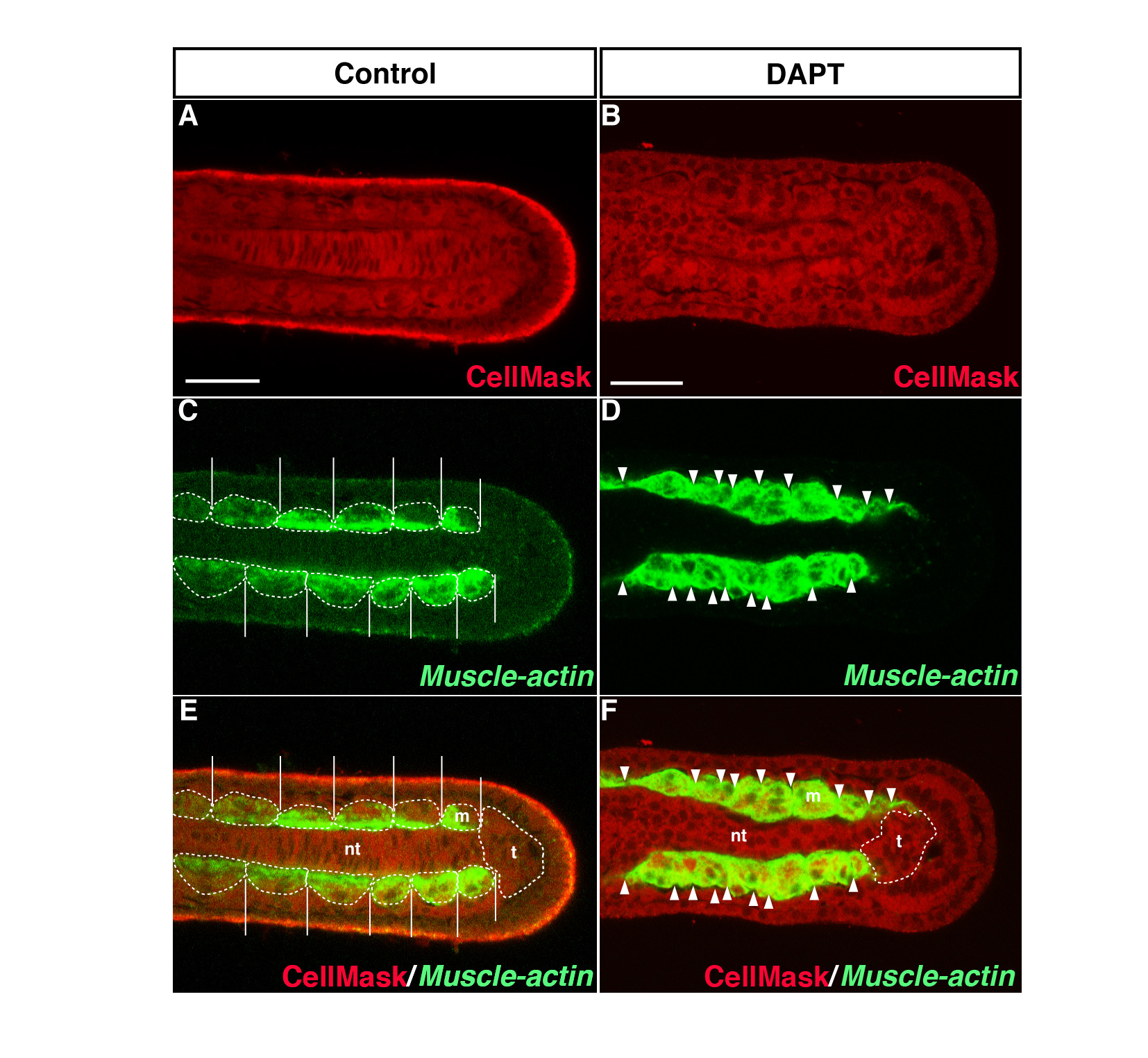


Supplemental Figure S2. Inhibition of Notch signalling and the effect on posterior myotomes at the early larva stage. (A, C, E) In the DMSO-treated control embryos, myotomes lay laterally to the notochord, and Bf*Muscle-actin* was expressed in the myotomes (n = 5, 100%) (B, D, F) In the DAPT-treated embryos, Bf*Muscle-actin* expression was detected in the myotomes (n = 5, 100%). The while line in (E) indicates the border of each myotome. The white dotted circle in (E) and (F) indicates a myotome or tail bud. The white arrowheads in (F) indicate a possible boundary between myotomes. m, myotome; nt, notochord; t, tail bud. Scale bars, 20 μm.
